# Institutional Questions

**Published:** 1900-04-07

**Authors:** 


### INSTITUTIONAL QUESTIONS.

**Accommodation for Cases of Infectious Disease.**

"Suburbia" writes: What steps can be taken in a district where, in spite of urgent representations on the part of some of the inhabitants, the Sanitary Authority cannot be induced to provide a sufficiency of accommodation for infectious cases, so that during an outbreak of scarlet fever no choice is left but for the sick to remain in their homes, a course obviously fraught with great danger to the community? Is not a proportion of 18 beds for isolation purposes to some 30,000 inhabitants far too small?

*** Probably the only way to induce the local authorities to realise their duties and responsibilities in this important matter is to bring pressure to bear on the members of the body constituting that authority—in the case in point the Urban District Council. Incidentally it should be pointed out that if people generally would remember their responsibilities as citizens, and take the trouble to secure the best representatives of the public interest on these local councils, these would not be the corrupt, inefficient, and unsatisfactory institutions they at present too often are. The proportion of beds you mention is of course inadequate, and every effort should certainly be made to bring the public authorities to see how foolish a policy they are pursuing in not making adequate provision for the proper isolation of infectious cases. No definite rules as to the proportion of beds to population are enforced by the L.G.B., but a representation to this central authority might do good.

